# Improvement of Methodology for Manual Energy Intake Estimation From Passive Capture Devices

**DOI:** 10.3389/fnut.2022.877775

**Published:** 2022-06-22

**Authors:** Zhaoxing Pan, Dan Forjan, Tyson Marden, Jonathan Padia, Tonmoy Ghosh, Delwar Hossain, J. Graham Thomas, Megan A. McCrory, Edward Sazonov, Janine A. Higgins

**Affiliations:** ^1^Department of Pediatrics, University of Colorado Anschutz Medical Campus, Aurora, CO, United States; ^2^Department of Biostatistics and Informatics, Colorado School of Public Health, University of Colorado Anschutz Medical Campus, Aurora, CO, United States; ^3^Colorado Clinical and Translational Sciences Institute, University of Colorado Anschutz Medical Campus, Aurora, CO, United States; ^4^Department of Electrical and Computer Engineering (ECE), The University of Alabama, Tuscaloosa, AL, United States; ^5^Department of Psychiatry and Human Behavior, Alpert Medical School of Brown University, Providence, RI, United States; ^6^Department of Health Sciences, Boston University, Boston, MA, United States

**Keywords:** passive device, energy intake, dietary analysis, food record, photograph, best practices, reproducibility

## Abstract

**Objective:**

To describe best practices for manual nutritional analyses of data from passive capture wearable devices in free-living conditions.

**Method:**

18 participants (10 female) with a mean age of 45 ± 10 years and mean BMI of 34.2 ± 4.6 kg/m^2^ consumed usual diet for 3 days in a free-living environment while wearing an automated passive capture device. This wearable device facilitates capture of images without manual input from the user. Data from the first nine participants were used by two trained nutritionists to identify sources contributing to inter-nutritionist variance in nutritional analyses. The nutritionists implemented best practices to mitigate these sources of variance in the next nine participants. The three best practices to reduce variance in analysis of energy intake (EI) estimation were: (1) *a priori* standardized food selection, (2) standardized nutrient database selection, and (3) increased number of images captured around eating episodes.

**Results:**

Inter-rater repeatability for EI, using intraclass correlation coefficient (ICC), improved by 0.39 from pre-best practices to post-best practices (0.14 vs 0.85, 95% CI, respectively), Bland–Altman analysis indicated strongly improved agreement between nutritionists for limits of agreement (LOA) post-best practices.

**Conclusion:**

Significant improvement of ICC and LOA for estimation of EI following implementation of best practices demonstrates that these practices improve the reproducibility of dietary analysis from passive capture device images in free-living environments.

## Introduction

Self-report methods of dietary intake such as written diet diaries, food frequency questionnaires, and photographic food records are commonly used to estimate dietary intake. Photographic food records are beneficial in their ease of use for participants compared to traditional written diet diaries, do not rely on recall by the participant, and have been validated for accuracy (1–5). Photographic food records typically contain two images, a pre- and post-ingestion photograph of the meal, taken by the participant at the correct distance, angle, and observing any other instructions to obtain clear photographs, such as cutting sandwiches in half to display fillings clearly or including a fiducial marker in the image as a size marker (6, 7). Though photographic food records have been shown to reduce participant burden in comparison to written diet diaries (4, 7, 8), there are still challenges associated with them, such as being able to identify specific food brands, nutritional claims (e.g., fat free, low sodium), and preparation methods in pre- and post-ingestion images with no other visible details (9). Passive capture methods may overcome some of these challenges by capturing multiple images at predetermined time intervals, without any action from the participant (10–14).

Emerging technologies present the opportunity to enhance dietary intake estimation by passively measuring eating activity in free-living settings with minimal user interaction. Collecting near-continuous data in the context of daily life to observe eating behavior and dietary intake has proven extremely difficult for researchers and burdensome for participants (15, 16). Wearable sensors can lessen the burden by passively collecting data while users go about their daily lives. Technological advances in dietary assessment tools include: (i) web-based self-administered 24-h recall tool, which aims to reduce respondent burden (17); (ii) mobile device-assisted ecological momentary assessment (mEMA), which focuses on reducing recall bias by collecting real-time data in free-living environments (18), (iii) photo-assisted and image-based dietary assessments, which attempt to reduce respondent burden and recall bias (19), and (iv) wearable sensors, which offer a suite of measurement tools that seek to tackle the limitations of other methods (15, 16).

Compared to photographic food records, where only two photos, one before and one after a meal, need to be analyzed by a trained nutritionist, passive capture methods necessitate viewing dozens of images at each eating episode to find the clearest image/s for analysis and including images of food preparation and/or packaging information, which increases nutritionist analysis time and cost.

The aim of this analysis was to document common sources of variation during manual nutritional analysis of passive capture diet records in free-living conditions and establish best practices for these analyses.

## Methods

### Participants

This was a multi-site study. Data were collected at Brown University and Boston University, the University of Alabama processed the passive capture data, and nutritional analysis was carried out by the Colorado Clinical and Translational Sciences Institute (CCTSI) Nutrition Core. The first nine participants (five females) with a mean (± SD) age of 42 ± 6 years (range: 37–56 years) and body mass index (BMI) of 36.3 ± 5.0 kg/m^2^ (range: 29.6–42.2) were designated as the pre-best practices cohort. The next nine participants (five females) with a mean age of 48 ± 13 years (range: 24–62 years) and BMI 32.0 ± 8.7 kg/m^2^ (range: 28.3–37.0) were designated the post-best practices cohort. Pre-best practices participants were 50% Caucasian, 25% Hispanic or Latino, 12.5% Black or African American, and 12.5% Portuguese. Post-best practices participants were 62.5% Caucasian, 25% Hispanic or Latino, and 12.5% Black or African American. Individuals who smoked or with temporo-mandibular joint disease, dysphagia, or other difficulties chewing and/or swallowing were excluded from participation.

Written informed consent was obtained from all individuals prior to participation in the study. The University of Rhode Island Institutional Review Board (URI IRB) approved this study. Participants included in the study were asked to wear the Automatic Ingestion Monitor (AIM-2) (20) device for seven consecutive days under free-living conditions for at least 12 h per day during waking hours. The AIM-2 is a wearable passive capture device containing an accelerometer and an optical sensor monitoring activation of the temporalis muscle for eating detection. A machine learning algorithm running on the device detected eating in real time and triggered a wide-angle camera aligned with the eye gaze when eating was detected. Photographs were passively captured at 15 s intervals. Any item in the field of view was captured as an image, including foods, beverages, containers, food labels, food preparation, and non-food images. Not all images were complete (i.e., half of a plate was out of the field of view; partial food labels) and others contained information useful to nutritionists (e.g., images of cutlery drawers, backpacks). Images were reviewed by a single researcher at each site and any considered private were removed prior to storage and analysis (20).

### Selecting Days for Analysis

Three days of dietary intake were analyzed per participant. For each participant there was an *a priori* primary sequence of the three days for analysis (two weekdays and one weekend day). For this sequence, Friday was considered a weekend day (21). If any of the days in the primary sequence did not meet inclusion criteria of weartime and eating episodes, the nutritionists used the remaining sequence of days (two weekdays and two weekend days) to replace the day(s) that did not meet the criteria (even if all days were weekdays). To be considered complete for data collection, a day had to have a minimum of 8 h of AIM wear time and at least two eating episodes between midnight and 11:59 pm. If a total of 3 days that met inclusion criteria could not be reached following utilization of all four alternate days, the participant was defined non-compliant and excluded from analysis.

### Dietary Analysis

Analysis of diet records was performed using the AIM Image Annotation Software (version V4.4). Images around each computer-derived eating episode were captured from micro-camera on the AIM-2 device (20). Two nutritionists independently analyzed resultant images for each participant. All nutritionists in the CCTSI Nutrition Core undergo standardized training before analyzing dietary intake data from clinical studies (22). For analysis of passive capture device food records, nutritionists are required to have a ≥ 0.85 intraclass correlation coefficient (ICC) inter-operator reliability, and validation tests are repeated quarterly (23).

Each food shown in images for an eating episode was entered in the AIM software by trained nutritionists who estimated food type, ingredients, preparation method (e.g., frying vs baking), and consumed amounts. Nutritionists used the United States Department of Agriculture (USDA) nutrient database (24) as the default because it contains nutritional information for the largest number of food items. This is an important consideration in free-living situations. If a food was not found in the USDA database, the Food and Nutrient Database for Dietary Studies (FNDDS) (24, 25) or the Branded Food Products Database (BFPD) (26) database was searched to find the best match for the food item.

### Identification of Common Sources of Error

**FIGURE 1:**
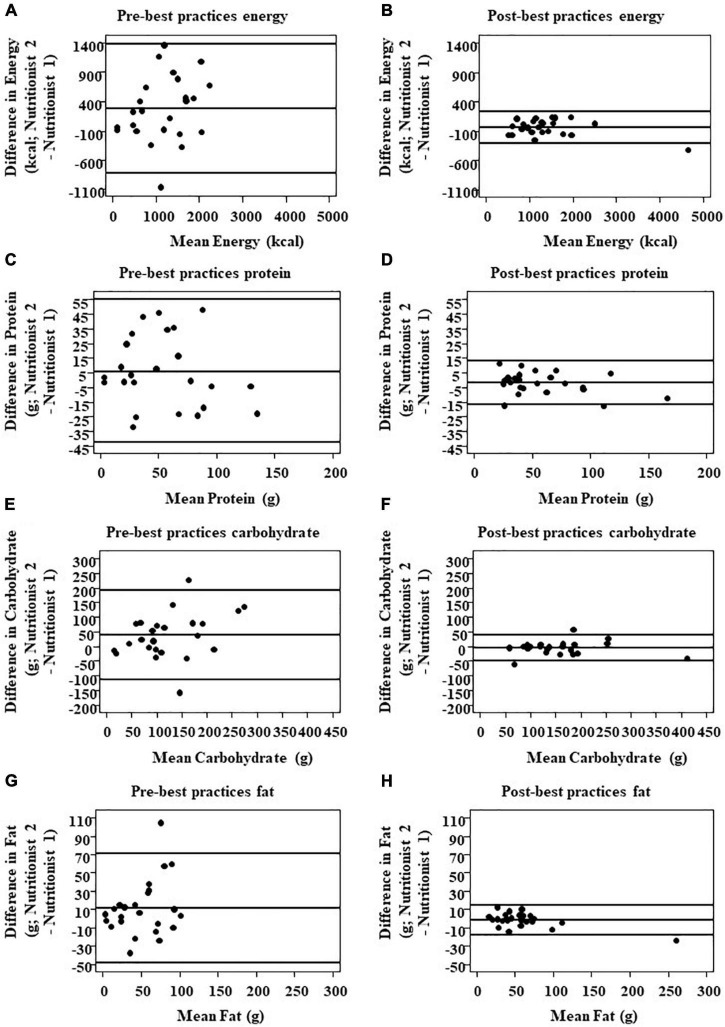
Comparison of inter-nutritionist agreement for total energy and macronutrient intake pre- and post-best practices implementation. Total energy **(A,B)**, protein **(C,D)**, CHO **(E,F)**, and fat intake **(G,H)** were compared via Bland–Altman plots (*n* = 24 pairs of measures assessed by two different nutritionists). The solid line in the center indicates 100% agreement, i.e., zero difference between nutritionists. The outer reference lines represent the upper and lower bound limits of agreement (LOA).

Initial comparison of analyses between independent nutritionists using the pre-best practices cohort (*n* = 8, as two participants did not have compliant weartime) showed large variance in daily energy intake (EI; Figure 1). After analysis, nutritionists, as a group, reviewed the statistical results from the pre- best practices cohort and identified three common occurrences that led to the large variance in EI, macronutrients, sodium, and fiber estimation: (1) inconsistency of energy and nutrient values between nutrient databases for the same food (e.g., one slice of a 14″ cheese thin crust pizza), (2) the default USDA database not providing detailed food preparation options, and (3) capture of an insufficient number of images of eating episodes to observe and analyze the eating episode (average of 5 ± 0.23 images/eating episode).

### Implementation of Best Practices

To resolve these issues, nutritionists conducted ten 60-min training analyses with all three nutritionists analyzing 56 days of eating episodes from the first eight (8) participants. During the training sessions, nutritionists determined the following best practices were necessary to improve analysis of passive capture images and reduce variability among nutritionists: (1) *a priori* standardization of food selection for commonly consumed, high energy density foods, (2) re-evaluating nutrient database hierarchy when analyzing images, and (3) reprogramming the AIM-2 to capture a higher number of images around eating episodes The group training sessions also established a protocol for food items that could not be easily identified. Nutritionists continuously bring items they cannot identify to the entire group to discuss on a regular basis. The food record is first marked as incomplete, and photos of the eating episode in question are researched and discussed by all nutritionists and the director. Once a solution is agreed upon, this food item is added to the standard operating procedure (SOP) for future reference. Instead of a nutritionist forming a conclusion independently on a unique food item, this practice prevents drift and keeps all nutritionists aligned for data entry practices over time.

#### Standardizing Food Selection

By selecting the same food and serving size but using different nutrient databases, USDA vs FNDDS for example, nutritionists can attain a large difference in daily EI. For example, using the same measure of a food (one slice = 1/8 of a 14″ thin-crust cheese pizza), there is a 202.4 kcal difference between the highest and lowest choice for exactly the same food and serving size (Supplementary Table 1). So, if a participant consumed a slice of pizza without showing the pizza box, each nutritionist might select a different choice from the nutrient database, which increases inter-nutritionist variance, even when choosing exactly the same consumed amount and type of food. For foods like pizza, the large variance in energy values between choices is exacerbated by the fact that more than one slice is usually consumed at each meal. Because of the EI differences caused by variance in choice, we assigned *a priori* choices for entry of commonly consumed high energy density foods. In the pre-best practices analysis, pizza was consumed by six of eight participants, hamburgers/cheeseburgers by four of eight, chicken nuggets by five of eight, chicken strips/tenders by four of eight, and tater tots by four of eight, so those foods were included the resultant *a priori* food selection guide (Supplementary Table 2).

Foods were grouped by type in this guide. For example, all 14″ thin crust cheese pizza choices were grouped. The *a priori* choice for this food item was designated by averaging the energy for each choice in the group and selecting the item closest to the mean. For example, the mean energy content of the 14″ thin crust cheese pizza slices was 244 kcals, and the closest item among all nutrient databases was Pizza Hut 14″ Thin N Crispy Crust pizza at 242 kcals/14″ slices. This was then selected as the default option for any slice of thin crust cheese pizza, regardless of if the brand was visible in the photo or not (Supplementary Table 2).

#### Nutrient Database Hierarchy

The AIM Image Annotation Software includes the USDA, FNDDS, and BFPD databases. The Standard Reference USDA Food Database contains over 8,000 food items, including foods and beverages that are primarily consumed by minority groups which provides easier food selection in free-living conditions although lacking the specific cooking and other food preparation methods contained in the FNDDS database (24). The FNDDS database contains over 7,000 food items and approximately 30,000 portion weights, as data from the National Health and Nutrition Examination Survey (27).

Published by the USDA Agriculture Research Service, the BFPD Database contains many commonly purchased branded and store branded items, such as Tyson chicken products, Stouffers frozen meals, and Wal-Mart store branded foods (26). The strength of this database is that store-bought brands are registered in the database, so it allows for very specific selection of branded foods that may not be available in other nutrient databases.

Access to multiple reference databases, each with multiple choices for a single food item, creates a plethora of choices for one specific food item. Therefore, two nutritionists can both pick the same food item and serving size but have a large variance in EI. For example, in the BFPD, there are over 80 choices for chicken nuggets. One nutritionist could choose Tyson breaded chicken nuggets (one nugget) and a second nutritionist could choose Applegate Farms chicken nuggets (one nugget). The difference in calories between the two chicken nugget brands is 23.4 kcal per nugget. This difference is compounded by the fact that more than one chicken nugget is consumed per meal.

Pre-best practices, the USDA database was selected as the default nutrient database because it contains the highest number of individual food items. During best practices implementation, the FNDDS database was chosen as the default because it includes more detailed choices for cooking and preparation options that can affect the energy content of food items. For example, for chicken, whether it is coated or not, whether skin is consumed, or the actual item was baked vs fried can markedly alter the energy content. Although this is estimated by each nutritionist for USDA food item entry, specific choices that include these options in the FNDDS database help to reduce inter-nutritionist variability.

To determine if switching to the FNDDS database would affect EI, nutritionists reanalyzed 31 pre-best practices food items by entering the exact same item in the FNDDS database compared to original item in the USDA or BFPD databases (Supplementary Table 3). For example, the USDA database for scrambled eggs is listed as “two large cooked scrambled eggs” and the FNDDS database is listed as “egg omelet or scrambled egg with no specification as to fat added in cooking.”

#### Increase of Images Around Eating Episodes

Pre-best practices implementation, the average images/eating episode was 5 ± 0.23 (*n* = 88 total eating episodes). This did not provide enough images for nutritionists to see definitive before and after eating images and or to see when participants made multiple reaches into opaque food containers such as a potato chip bag. The AIM was reprogrammed by engineers at the University of Alabama to capture more images during and around eating episodes and, for post-best practices implementation, the average number of images/eating episode increased to 11 ± 0.41 (*n* = 92 total eating episodes).

The higher number of images during eating episodes allowed nutritionists to see food preparation methods, ingredients, brand names, and, after the meal, leftovers, desserts, or smaller single sips or bites during clean up that were not previously seen in pre-best practices images. This is beneficial when participants consume mixed dishes, such as soups, salads, or sandwiches so that all the ingredients and condiments, not easily visible in a photo of the food as consumed, can be identified and quantitated.

More images during eating episodes facilitates observation of the actual amount of food consumed, particularly when a participant is snacking from a container or bag and is advantageous for identifying liquid in an opaque mug or container. Being able to view more images of the beverage and its preparation, nutritionists can see important details such as whether sugar or zero calorie sweetener was added, or the type of milk in tea or coffee.

### Statistical Analysis

Separate analysis was conducted for pre- and post-best practices cohorts, respectively. Bland–Altman analysis was used to descriptively and graphically describe the agreement of measures by two different nutritionists. The SD of the between-nutritionist difference was estimated by a linear mixed model with the individual difference as the dependent variable, intercept as the independent variable, and a compound symmetry covariance structure to account for the correlation of repeated measures of the same participant on three different days. The percent difference in estimates of EI for the database selection for the same food items before and after the best practices was tested using a two-sided *t*-test. The percent difference analysis was the only analysis done on single food items, all other analyses were performed for daily intake. Inter-rater reliability was evaluated using ICC (Table 1). Inter-rater repeatability was assessed using repeatability coefficient (RC; Table 2). RC is defined as 1.96 × $\sqrt{2}$ × (within-subject SD). ICC and RC were all estimated based on the linear mixed effects model with the outcome assessed by either nutritionist as dependent variable and an intercept only as the independent variable as well as a compound symmetry structure to model the variance of two measures assessed by two nutritionists. Using 5,000 bootstrap samples and the percentile method, the 95% confidence interval for ICC and RC at pre- and post-best practice implementation and the post-best practice change were estimated. Statistical inferences for the post-best practices change in ICC and RC were made based on the confidence interval. Followed Landis and Koch (28), ICC was interpreted as ≤ 0 (poor), 0.0–0.2 (slight), 0.2–0.4 (fair), 0.4–0.6 (moderate), 0.6–0.8 (substantial), and 0.8–1.0 (excellent). The change was deemed to be statistically significant (*p* < 0.05) if the 95% CI did not include zero. SAS 9.4 (SAS institute, Cary, NC) was used for all analyses.

**TABLE 1:** Intraclass correlation coefficients (95% confidence interval) between operators.

Outcome	Pre-best practices	Post-best practices	Difference in intraclass correlation coefficient (95%) CI between two methods^1^
Total energy (kcal)	0.6 (0.14, 0.83)	0.99 (0.92, 0.99)	0.39 (0.14, 0.85)*
Carbohydrate (g)	0.79 (0.14, 0.91)	0.98 (0.95, 0.99)	0.18 (0.07, 0.85)*
Fat (g)	0.56 (0.23, 0.81)	0.99 (0.94, 0.99)	0.43 (0.14, 0.75)*
Protein (g)	0.44 (−0.05, 0.68)	0.96 (0.86, 0.98)	0.52 (0.25, 0.98)*
Fiber (g)	0.09 (−0.06, 0.56)	0.91 (0.82, 0.97)	0.82 (0.35, 0.98)*
Sodium (mg)	0.36 (−0.02, 0.72)	0.92 (0.80, 0.97)	0.56 (0.17, 0.94)*

*^1^95% CI is based on 5,000 bootstrap analysis.*

*p < 0.05.

**TABLE 2:** Repeatability coefficients (95% confidence interval) between operators.

Outcome	Pre-best practices	Post-best practices	Difference in repeatability coefficient (95%) CI between two methods^1^
Total energy (kcal)	1205.6 (813.4, 1512.6)	281.5 (201, 383.4)	−924.0 (−1302.6, −551.9)*
Carbohydrate (g)	167.8 (122.7, 222.5)	44.1 (28.0, 55.7)	−123.6 (−167.7, −78.8)*
Fat (g)	63 (38.2, 89.4)	15.6 (10.2, 21.3)	−47.4 (−75.9, −19.8)*
Protein (g)	48.3 (35.7, 60.1)	15 (9.7, 18.9)	−33.3 (−43.9, −18.4)*
Fiber (g)	31.2 (11.7, 46.9)	4.9 (2.2, 6.5)	−26.3 (−42.4, −6.8)*
Sodium (mg)	2361.8 (1579.1, 3140.5)	1213.2 (641.0, 1707.9)	−1148.7 (−2196.2, −135.6)*

*^1^95% CI is based on 5,000 bootstrap analysis.*

*p < 0.05.

## Results

Of 18 participants, 16 were used for three-day passive capture nutritional analyses. Two participants were excluded because they did not have enough days of compliant AIM wear time.

For every dietary outcome measure, daily EI and macronutrient intake, implementation of best practices improved the limits of agreement (LOA) between nutritionists (Figure 1). The post-standardization LOA was remarkably narrower as compared to the LOA prior to standardization. LOA for EI was −278.4 ± 114.4 kcal/d pre-best practices and −39.5 ± (32.9) kcal/d post-best practices; carbohydrates 38.6 ± (15.9) g/d pre-best practices and −4.3 ± (4.6) g/d post-best practices; fat 11.5 ± (6.3) g/day pre-best practices and −1.6 ± (2.1) g/day post-best practices; protein 6.1 ± (7.1) g/day pre-best practices and −1.9 ± (1.5) g/day post-best practices; fiber 2.4 ± (3.3) g/day pre-best practices and −0.4 ± (0.5) g/day post-best practices; and sodium 484.5 ± (230.1) pre-best practices and −168.4 ± (124.2) post-best practices.

Interrater reliability (Table 2) as assessed by ICC improved significantly for daily total EI (Δ, 0.39 (95% CI: 0.14, 0.85), protein (Δ = 0.18;95% CI: 0.07, 0.85), fat (Δ = 0.43; 95% CI:0.14, 0.75), carbohydrate (Δ = 0.52; 95% CI: 0.25, 0.98), fiber (Δ = 0.82; 95% CI: 0.35, 0.98), and sodium intake (Δ = 0.56; 95% CI: 0.17, 0.94). The post-standardization reliability as assessed by ICC was in the excellent range (ICC ≥ 0.90) for every measured outcome whereas pre-standardization ICCs varied between the poor and substantial categories. Similarly, significant improvement in repeatability coefficients were seen for all the outcome measures (Table 2).

Post-best practice implementation, nutritionists used the *a priori* food selection guide 100% of the time when a food item appeared in an eating episode.

The difference in EI for nutrient database hierarchy pre- and post-best practices implementation, that is, defaulting to the FNDDS vs USDA, is 64.4kcal ± 91.2.2kcal (40%; Supplementary Table 3). Supplementary Table 3 shows the percent difference in the same foods entered similarly into different databases. The use of the two databases for the same foods highlights the possible variation when database selection is not standardized. The initial energy measure represents the use of the USDA nutrient database as the default. In the *a priori* food selection guide were excluded from this Figure.

## Discussion

To our knowledge, this is the first study that has sought to implement best-practices for manual nutritional analysis of passive image capture images in free living conditions. Gemming et al. (29) discussed that wearable cameras offer the opportunity for secondary analysis, increasing the usefulness of data obtained but does not detail what training or best practices can be implemented.

Previous studies that use passive methods to estimate EI and macronutrient composition in free-living environments have encountered similar challenges to this study. Cooking methods that affect EI and nutrient composition in passive image capture methods are a common obstacle (30), as are low quality images that make it difficult for nutritionists to accurately estimate EI (8, 29, 31, 32). Beltran et al., Schrempft et al., and Martin et al. showed low ICC between nutritionists for portion size estimates in free-living conditions (32–34) as opposed to other traditional digital photography methods (35).

For analysis of passive capture device food records, CCTSI Nutrition Core nutritionists are required to have a 0.85 ICC inter-operator reliability which is repeated quarterly (23). However, images used for training and ongoing competency assessments were from AIM-acquired images in a lab setting, not in a free-living environment. All food in these images came from a university cafeteria with a limited menu rotation. Additionally, participants consumed food at a table in a well-lit environment, and nutritionists could easily identify which food items the participants consumed. In a free-living environment, participants have exponentially more food choices, preparation methods, are not eating in a controlled environment, and have food/beverages in their vicinity that may or may not be part of their meal, especially when eating in a communal or family setting. The lab-setting passive device food images are useful to help nutritionists navigate the analysis software and to analyze food photographs at different angles and in different light conditions than traditional photographic food records, but implementing additional best practices is necessary to obtain reproducible results in a free-living environment.

The *a priori* food selection guide reduced variability in all outcome measures for high energy density, commonly consumed food items as nutritionists selected the same choice consistently. Many participants consumed non-branded food items from local stores or restaurants, so having *a priori* food choices minimizes overall bias between participants. By using the *a priori* food selection guide, nutritionists can independently select the correct food within the database, without searching for an appropriate brand, selecting a brand that is not listed, or decide best fit on a per-nutritionist basis. It should be noted that the *a priori* food selection guide will evolve over time. As analysis continues with passive device images, nutritionists will identify more items that are deemed to need *a priori* selections.

Following best practices implementation, nutritionists used the FNDDS database as the default when searching for and selecting foods for analysis, unless otherwise indicated by the *a priori* food selection guide or if a food is not available in FNDDS. For example, the Pizza Hut 14″ thin crust cheese pizza slice is found in the USDA database, and is the *a priori* choice for any thin crust cheese pizza.

The advantages of using an *a priori* food selection guide for nutritional analysis may depend on the nature of the research. Using *a priori* food selections and defaulting to the FNDDS database may contribute to small inaccuracies of EI and macronutrient intake at the participant level, but these are minimized at the group level. For individual data, such as providing personalized, real time dietary intake feedback during weight loss, using the *a priori* food selection guide as a default may not be appropriate as one would require accurate data for that individual. For group level data, such as a randomized, controlled dietary intervention study, using the *a priori* food selection guide would be appropriate as the data would be reproducible for the overall group or population and, when data are presented as means, small differences in accuracy between participants will be minimized (36, 37).

Increasing the frequency of images taken around eating episodes to include meal or snack preparation methods allowed nutritionists to both see the ingredients in mixed dishes, such as soups and salads, and the cooking methods of foods such as meats and eggs. Pre-best practices, a certain amount of estimation was involved but additional photographs that show preparation and cleanup helps to mitigate assumptions by providing more opportunity to view clear images for analysis. The limitations to the addition of more images is shorter device battery life and an increased amount of data storage necessary to capture and store all images.

In addition to food preparation information, increased frequency of images also gives more opportunity to identify foods in opaque containers, low light settings, or any instance where the camera may be partially obscured, such as by clothing or hair. With a wearable capture device, participants are also often in motion, resulting in blurry or partially obscured images, without the full range of food items visible in a single image (8, 29, 31, 32). More images provides nutritionists with a higher likelihood of identifying a clear picture that contains all or most foods.

Implementation of database standardization, the *a priori* food selection guide, and image frequency improved LOA between nutritionists greatly. The percent difference for LOA for EI pre- and post- best practices was 150.3%, carbohydrates 159.9%, protein 105%, and fats 151.1%, indicating significant difference after best practices implementation. All post-best practice numbers contained a much narrower limit of agreement, indicating that implementing best practices for this dataset decreased inter-nutritionist variability in energy and macronutrient intake for these analyses.

The main strengths of the study were that images were captured in a free-living environment, use of experienced nutritionists trained in photographic dietary intake assessment, and the diversity of the participants. Limitations of this study include the relatively small sample size, an unequal number of eating episodes between pre- and post-best practice implementation, and that no weighed food records or doubly-labeled water was used in this study so, although reproducibility was measured, we were unable to measure accuracy. Future investigation in a variety of free-living environments is necessary to determine the effects of these best practices on the accuracy of manually estimating dietary intake from passive capture devices.

## Conclusion

Implementation of three best practices: standardizing food selection, nutrient database hierarchy, and increase of images around eating episodes, improves the reproducibility of manual dietary analysis of images from wearable passive capture device images.

## Data Availability Statement

The original contributions presented in this study are included in the article/Supplementary Material, further inquiries can be directed to the corresponding authors.

## Ethics Statement

The studies involving human participants were reviewed and approved by the University of Rhode Island Institutional Review Board. The patients/participants provided their written informed consent to participate in this study.

## Author Contributions

DF, TM, ES, MM, and JH: conceptualization. DF, TM, JP, JH, ES, MM, JT, TG, and DH: data curation. DF and ZP: investigation. DF, TG, and DH: project administration. ES, MM, JH, and JT: supervision and writing. DF, TM, ZP, JP, and JH: original draft. DF, ZP, TM, JP, TG, DH, JT, MM, ES, and JH: writing – review and editing. All authors contributed to the article and approved the submitted version.

## Author Disclaimer

The content is solely the responsibility of the authors and does not necessarily represent the official views of the National Institutes of Health.

## Conflict of Interest

The authors declare that the research was conducted in the absence of any commercial or financial relationships that could be construed as a potential conflict of interest.

## Publisher’s Note

All claims expressed in this article are solely those of the authors and do not necessarily represent those of their affiliated organizations, or those of the publisher, the editors and the reviewers. Any product that may be evaluated in this article, or claim that may be made by its manufacturer, is not guaranteed or endorsed by the publisher.

## Supplementary Material

Supplementary Table 1 Comparison of possible entries for commonly consumed foods.

Supplementary Table 2 *A priori* food selection guide.

Supplementary Table 3 Difference in energy values of the same food from different UDSA vs FNDDS nutritional databases.
